# Identification of the Functional Variant(s) that Explain the Low-Density Lipoprotein Receptor (*LDLR*) GWAS SNP rs6511720 Association with Lower LDL-C and Risk of CHD

**DOI:** 10.1371/journal.pone.0167676

**Published:** 2016-12-14

**Authors:** Roaa Hani Fairoozy, Jon White, Jutta Palmen, Anastasia Z. Kalea, Steve E. Humphries

**Affiliations:** 1 Centre for Cardiovascular Genetics, BHF Laboratories, Institute of Cardiovascular Science, University College London, London, United Kingdom; 2 University College London Genetics Institute, Department of Genetics, Environment and Evolution, London, United Kingdom; University of Miami School of Medicine, UNITED STATES

## Abstract

**Background:**

The Low-Density Lipoprotein Receptor (*LDLR*) SNP rs6511720 (G>T), located in intron-1 of the gene, has been identified in genome-wide association studies (GWAS) as being associated with lower plasma levels of LDL-C and a lower risk of coronary heart disease (CHD). Whether or not rs6511720 is itself functional or a marker for a functional variant elsewhere in the gene is not known.

**Methods:**

The association of *LDLR* SNP rs6511720 with incidence of CHD and levels of LDL-C was determined by reference to CARDIoGRAM, C4D and Global lipids genetics consortium (GLGC) data. SNP annotation databases were used to identify possible SNP function and prioritization. Luciferase reporter assays in the liver cell line Huh7 were used to measure the effect of variant genotype on gene expression. Electrophoretic Mobility Shift Assays (EMSAs) were used to identify the Transcription Factors (TFs) involved in gene expression regulation.

**Results:**

The phenotype-genotype analysis showed that the rs6511720 minor allele is associated with lower level of LDL-C [beta = -0.2209, p = 3.85 x10^-262^], and lower risk of CHD [log (OR) = 0.1155, p = 1.04 x10^-7^]. Rs6511720 is in complete linkage. Rs6511720 is in complete linkage disequilibrium (LD) with three intron-1 SNPs (rs141787760, rs60173709, rs57217136). Luciferase reporter assays in Huh7 cells showed that the rare alleles of both rs6511720 and rs57217136 caused a significant increase in *LDLR* expression compared to the common alleles (+29% and +24%, respectively). Multiplex Competitor-EMSAs (MC-EMSA) identified that the transcription factor serum response element (SRE) binds to rs6511720, while retinoic acid receptor (RAR) and signal transducer and activator of transcription 1 (STAT1) bind to rs57217136.

**Conclusion:**

Both *LDLR* rs6511720 and rs57217136 are functional variants. Both these minor alleles create enhancer-binding protein sites for TFs and may contribute to increased *LDLR* expression, which is consequently associated with reduced LDL-C levels and 12% lower CHD risk.

## Introduction

Elevated plasma lipid levels promote atherosclerosis and increase the risk of coronary heart disease (CHD). Low-density lipoprotein cholesterol (LDL-C) is taken up from the blood by the LDL-Receptor (LDL-R). *LDLR* is located on chromosome 19 at p13.1-p13.3 and it encodes a cell surface glycoprotein predominantly expressed in hepatocytes. LDL-R mediates the removal of cholesterol-carrying LDL-C particles from the blood via ApoB-100 [1–3]. The 45kb gene comprises 18 exons and 17 introns [4]. Mutation in the *LDLR* gene leads to the monogenic disorder, familial hypercholesterolemia (FH), and to date over 1,200 mutations have been reported in the *LDLR* gene that cause FH [5]. The vast majority of these mutations are located in the exonic regions, and thus affect protein structure and function, while 10% are in the intronic region (exon boundary), and these are predicted to affect correct splicing, and 2% in the promoter region, which are predicted to prevent gene transcription. A single nucleotide polymorphism (SNP) within *LDLR* exon 12, rs688 is associated with both LDL-C and CHD in a gender-independent mode [6, 7]. It also acts as a modulator of alterative exon splicing, which can lead to a shift in the reading frame and an altered gene transcript [8–11]. Non-coding SNPs in *LDLR* have also been reported to be functional, for example, in the promoter region c.-139C>G [12], c.-101T>C, c.-121T>C [13], and -49C>T [14], and rs17248720 in the intergenic region [15] are involved in regulation of gene expression and have been reported to cause FH.

In the last decade, genome-wide association studies (GWAS) have identified numerous loci that harbor common signal nucleotide polymorphisms (SNPs) which have relatively small effects on lipid traits, including at the *LDLR* locus where SNPs are associated with LDL-C levels. The majority of common variants that have been discovered in GWAS are in non-coding regions and their functional implications are unknown [16]. Interpretation of the molecular mechanisms of non-coding variants is a huge challenge because of linkage disequilibrium (LD) and the diversity of non-coding functions, including transcriptional, mRNA splicing and control of translation [17, 18]. The T allele of the *LDLR* SNP rs6511720 (G>T) [MAF = 0.10 in a European population, (1000 Genomes Project Phase 3)] has been identified as being associated with lower plasma levels of LDL-C (size effect: -0.15 to -0.26 mmol/L) and a lower risk of CAD [19–21], myocardial infarction (MI) [22] and abdominal aortic aneurysm (AAA) [23]. Between-study similarities have provided confidence that the *LDLR* SNP rs6511720 is either functional or may be a marker for a functional variant elsewhere in the gene. In addition, Talmud et al. (2013) constructed a weighted LDL-C-raising gene score of 12 common LDL-C-raising SNPs previously identified by the Global Lipids Genetics consortium, including the *LDLR* SNP rs6511720 [19] in two patient groups (FH without an identified mutation, FH with an identified mutation) and one control group. They found that the mean weighted SNP score for both mutation-negative and mutation-positive FH patients was significantly higher than in control subjects. The difference between mutation-negative and mutation-positive also was significant. They proposed that these common LDL-C-raising SNPs explained the hypercholesterolemia phenotype in at least 80% of patients with a clinical diagnosis of FH but with no identified mutation [24], however, the functional roles of many of these SNPs are unknown.

The rs6511720 SNP is located in intron-1 of the *LDLR* gene, where *cis*-acting gene regulatory sites are commonly found [25]. *Cis*-regulatory elements physically interact with the promoter region of a gene to initiate DNA transcription [26–28]. Such SNP could be a part of an enhancer element due to a modification of the transcription factor-binding site (TFBS) that in turn recruits co-activators and chromatin regulators to facilitate the transcription of the *LDLR* gene [29]. However, the analysis of the genetic function of such variants is complex because of the LD between SNPs, which are co-inherited with a causal variant. Thus, all SNPs in LD with the functional variant may carry some or all of the associations with the trait of interest, although they have may have not any relevant function.

This study uses a range of methods to identify functional variants and their role in gene regulation. We employed SNP selection and prioritization methods based on the data from bioinformatics databases. Three SNPs (rs57217136, rs141787760 and rs60173709) in addition to GWAS hit SNP rs6511720 were selected for functional analyses. A number of functional study techniques were performed including conventional electrophoretic mobility shift assays (EMSA), reporter assay and multiplex competitor electrophoretic mobility shift assays (MC-EMSA) to identify the regulatory role of selected SNPs.

## Methods

### Statistical analysis

The summary estimates for the log odds Ratio (OR) and its standard error of rs6511720 on CHD were taken from CARDIoGRAM [30] and C4D [31] (http://www.cardiogramplusc4d.org/downloads/) and were combined by fixed effects meta-analysis. We obtained an estimate of the regression coefficient for rs6511720 on LDL-C from Global lipids genetics consortium (GLGC) [32] (http://csg.sph.umich.edu/abecasis/public/lipids2013/). All analyses were conducted using the statistical computing environment R version 3.2.0.

### SNP annotation

Several SNP annotation databases were used to identify possible SNP functions. The UCSC genome browser [33] was used as the source of genome-wide maps of the chromatin state of the region of interest of the gene. Locuszoom [34] (http://csg.sph.umich.edu/locuszoom/), SNAP [35] (https://www.broadinstitute.org/mpg/nsnap/ldplot.php) and HaploReg V2-V4 [36, 37] (http://compbio.mit.edu/HaploReg) were used to identify LD SNPs with the *LDLR* GWAS hit SNP rs6511720. HaploReg and MatInspector [38] (Genomatix Software GmbH, Germany) were used to create the SNP profile. Three SNPs in complete LD (r^2^ = 1) with GWAS hit SNP rs6511720 (rs57217136, rs141787760 and rs60173709) were selected for further analyses.

### Functional assessment of *LDLR* intron-1 SNPs

Electrophoretic mobility shift assays (EMSAs) were used to investigate the effect of variants’ genotype on DNA-protein binding. Probes of 31 bp sequences that encompassed the common or rare variant of each of the four SNPs were employed (probe sequences are available upon request). Probes were labeled using the biotin 3’-end DNA labeling kit (Pierce, Rockford, IL, USA) as recommended by the manufacturer. Each binding reaction consisted of 10μl of 2X binding buffer (16% Ficol, 40mM HEPES, 100mM KCl, 2mM EDTA, and 1mM DTT), 0.8ng of poly(dI.dC), 50mM of MgCl_2_, 0.8mg of BSA and 2nmol of Huh7 (a human hepatocellular carcinoma cell line) nuclear extract, corrected with dH_2_O to a final volume of 20μl. The reaction mixture was incubated at 25°C for 30 min, followed by the addition of 6X loading buffer. Samples were electrophoresed on a 6% polyacrylamide gel for 180–240 min at 120V and then transferred to a nylon membrane using Southern transfer. The images were obtained using a chemiluminescent nucleic acid detection module (Pierce, Rockford, IL, USA) according to the manufacturer’s instructions. Multiplex competitor electrophoretic mobility shift assay (MC-EMSAs) were carried out to identify the TFs that are involved in expression differentiation between alleles of rs6511720 and rs57217136 SNPs. The MC-EMSAs were performed using seven sets of cocktails, each with ten unlabeled dsDNA consensus sequences for well-characterized TFBS [39]. These TF cocktails were incubated with the binding reaction mix comprising Huh7 nuclear extract for 15 min and then a labeled probe was added and incubated at 25°C for 30 min. When a particular set of TFs eliminated the band shift, as a result of binding the unlabeled competitor, the individual competitor from this set was examined by an additional EMSA, in order to specify the TF that bound to the allele of the variant.

Luciferase reporter assays were performed to determine whether the four *LDLR* intron-1 SNPs influenced gene expression. The Phusion^®^ High-Fidelity PCR Kit (New England BioLabs Inc) was used to amplify DNA fragments of interest including the *LDLR* promoter (594bp), and the *LDLR* SNPs rs6511720 (883bp), rs57217136 (814bp), rs141787760, as well as rs60173709 (643bp), as recommended by the manufacturer (primer sequence available upon request).

For cloning, the In-Fusion^®^ HD Cloning Kit (Clontech Laboratories, Inc.) was used following the manufacturer’s instructions. The *LDLR* promoter DNA fragment was inserted into a pGL3-basic luciferase reporter vector to generate a LDLR**-**luciferase-construct (promoter only) reporter plasmid. The insertion was upstream of the Luciferase gene (*luc*+) at the promoter site. Then, the *LDLR* intron-1 SNP sequences encompassing the SNP allele were individually inserted into the enhancer site of the LDLR**-**luciferase-construct after the SV40 polyadenylation signal. These constructs were then transformed in *E-coli* cells.

Using a quikChange Site-Directed Mutagenesis kit (Stratagene, La Jolla, CA, USA), the rs6511720 G>T variant was created at position 79, rs141787760 (C>-) and rs60173709 (T>-) deletions were generated individually at positions 41 and 247, respectively, and rs57217136 C>T mutation was created at position 401. Both original and mutated LDLR**-**luciferase-constructs were transfected into Huh7 cells along with the pRL-TK plasmid as a renilla luciferase control reporter vector. When the Huh7 cells were 80% confluent, they were plated into a 96-well plate (2x10^4^ cells/well) and incubated for 24 hours. Transfection was carried out using Opti-MEM^®^ reduced serum medium and lipofectamine 2000 (Invitrogen). Luciferase activity was measured using the dual Luciferase Reporter Assay System kit (Promega), following the manufacturer’s instructions. The mean relative expression difference between variants’ genotype was determined by a two-sample *t*-test.

## Results

### Association of rs6511720 genotype with LD-C and CHD

The meta-analysis of the CARDIoGRAM and the C4D data showed that the rs6511720 minor allele was associated with lower risk of CHD [log (OR) = −0.1155; SE = 0.0217; p = 1.04 x10^-7^] (S1 Table). The GLGC (N = 170,607) showed that rs6511720 minor allele is associated with lower levels of LDL-C [beta = −0.2209; SE = 0.0061; p = 3.85 x10^-262^] (S2 Table). This suggests that individuals carrying one or more copies of the minor LDL-C lowering T allele of rs6511720 would be at lower lifetime risk of CHD.

### SNPs annotation and prioritization

**Fig 1:**
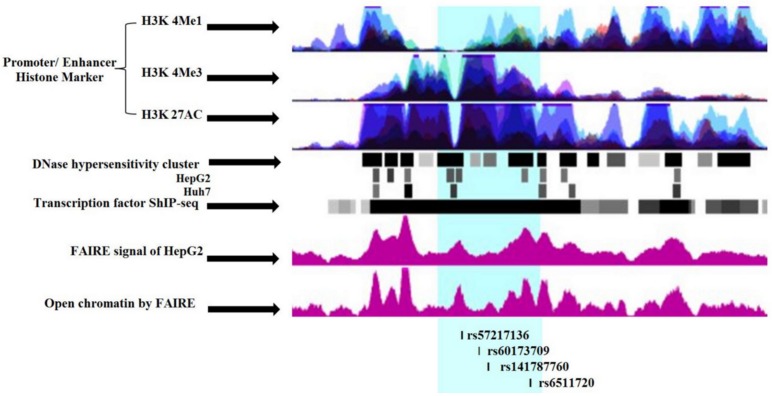
Genome-wide maps of chromatin state of LDLR intron-1. Schematic presentation of the LDLR intron-1 chromatin status (https://genome-euro.ucsc.edu). The area of interest in intron-1 is highlighted in light-blue color. Promoter/ Enhancer histone marker of seven cell lines (GM12878, H1-hESC, HSMM, HUVEC, K562, NHEK, and NHLF). FAIRE: formaldehyde assisted isolation of regulatory elements.

To identify potentially functional SNPs at the *LDLR* locus, we considered variants with strong LD (r^2^ = 0.8) with the *LDLR* GWAS hit SNP rs6511720. This SNP is in strong LD with 48 other SNPs: three SNPs are located in intron-1 and the others are located ≥1.5kb upstream of *LDLR* locus (see LD plot, S1 Fig). To further prioritize variants for a functional analysis follow-up, genome-wide maps of the chromatin state of relevant cell types (HepG2, human liver hepatocytes) were examined. Variant position was evaluated for evidence of the histone modification mark H3K4me1, H3K27Ac and H3K4me3, as well as for DNase I hypersensitivity sites and for formaldehyde assisted isolation of regulatory elements (FAIRE). The post-translational chromatin mark H3K4me1, and H3K27Ac are often associated with enhancer regions [40], while H3K4me3 associated with promoter regions [41, 42]. DNase I and FAIRE are established methods for the identification of nucleosome regulatory regions [43] (Fig 1). In addition, TFs occupancy in chromatin was assessed using genome wide ChIP-seq data sets (UCSC genome browser accessed 01-03-2016). Of the 48 variants meeting the LD threshold (r^2^<0.8), four SNPs were found to have strong chromatin signals and interesting TF binding profiles (S1 Table) including three SNPs in complete LD (r^2^ = 1) with rs6511720 (rs57217136 (T>C), rs141787760 (C>deletion) and rs60173709 (T>deletion). The four selected SNPs are located in intron-1 of *LDLR* within ≈ 1200bp of each other.

*In-silico* tools, MatInspector and HaploReg, showed different DNA-protein binding profiles of these SNPs (S1 Table). The MatInspector software identified a sequence around the minor allele of rs6511720 that was predicted to bind to GATA and the snRNA activating protein/ proximal sequence element (SNAP/PSE) complex, but HaploReg did not predict any protein binding around this SNP. SNAP/PSE has a role in gene transcription initiation [44–46]. MatInspector did not find any binding site for either rs141787760 and rs60173709, while HaploReg software predicted that the minor allele of rs141787760 would bind to TFs that have roles in chromatin modulation through general transcription regulation and a known *LDLR* transcription regulator SP1 (specificity protein 1). Rs60173709 was also predicted as a binding site for some proteins, but not any that have a clear role in *LDLR* gene regulation. For rs57217136 HaploReg predicted that the major allele would bind to TFs such as forkhead box protein A (FOXA1, FOXA2), which are liver transcription activators, and sterol regulatory element-binding protein 1 (SREBP1), while the minor allele would bind to SP1.

### Allele-specific protein binding of *LDLR* intron-1 SNPs in Huh7 cells

**Fig 2:**
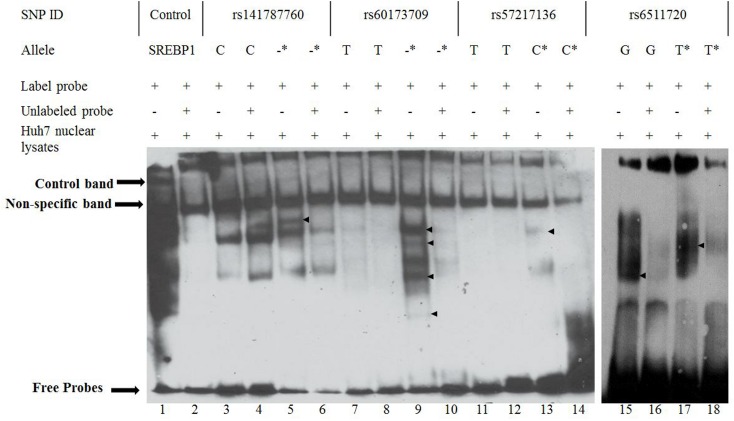
DNA binding properties of *LDLR* intron-1 SNPs. Conventional EMSA analysis of the *LDLR* intron-1 SNPs (rs6511720, rs141687760, rs60173709, and rs57217136). Binding of SREBP1 was used as the control (lane 1 and 2). The lanes with a labeled probe showed a specific band indicated by arrows, while when the unlabeled probe was added the band disappeared. These four SNPs have allele-specific binding, indicated by arrows. (-) = deletion and (*) = minor allele.

The *in-silico* analysis suggested that the *LDLR* intron-1 selected SNPs are sites for TF binding. To assess whether the alleles of the SNPs differentially affect protein-DNA binding *in vitro*, EMSAs for the four intron-1 SNPs were carried out using Huh7 nuclear lysates. As shown in Fig 2, all four SNPs demonstrated allele-specific binding. The rare alleles of rs57217136, rs141787760 and rs60173709 demonstrated a new DNA-protein complex, while the rs6511720 T allele formed a different protein complex, which moved more slowly than the major G allele complex.

### Allele-specific enhancer activity of *LDLR* intron-1 SNPs in Huh7 cells

**Fig 3:**
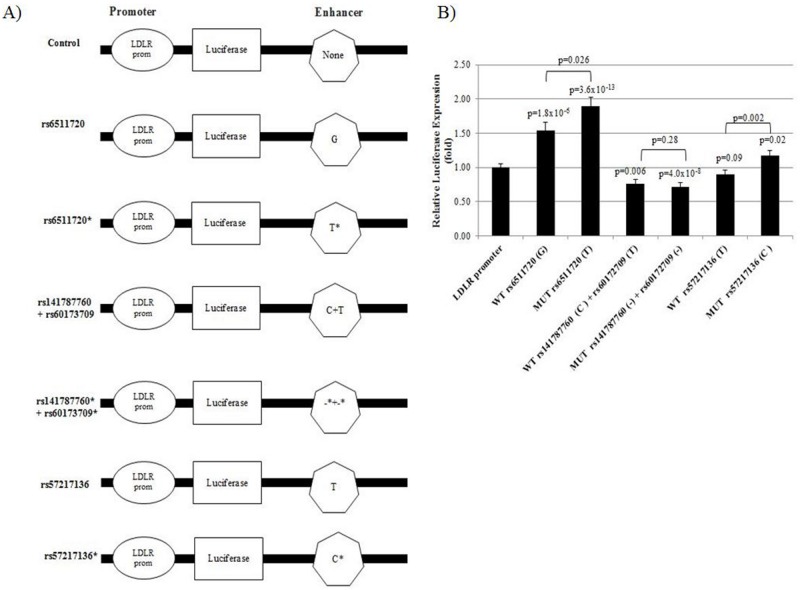
*LDLR* luciferase constructs and SNP luciferase activity in Huh7 cell line. A) Schematic presentation of LDLR**-**luciferase-construct (promoter only) and LDLR**-**luciferase-enhancer-constructs. The constructs were transfected into Huh7 cells. B) Results of luciferase reporter assays showing relative expression of LDLR-luciferase-enhancer constructs of *LDLR* SNPs relative to the LDLR-luciferase (no enhancer) construct. (-) = deletion and (*) = minor allele.

Luciferase reporter assays were carried out to determine the influence of the four SNPs on the transcriptional activity of the *LDLR* promoter. Generating a fragment comprising all four SNPs was not possible because intron-1 of the *LDLR* has 20 Alu repeats [47]. Therefore, three fragments were generated to exclude Alu repeats; the first fragment contained rs6511720, the second fragment rs141787760/rs60173709, and the third fragment rs57217136. These fragments were inserted individually into the enhancer site of the LDLR**-**luciferase-construct and transfected into Huh7 cells (Fig 3A). Luciferase activity measurements showed that the rs141787760/rs60173709 transfected construct led to a significant reduction in *LDLR* promoter expression when compared to the promoter alone: -24% (p = 0.006) for the major allele and -29% (p = 4.0x10^-8^) for the minor allele, but with no significant difference between the alleles. In contrast, the rare allele of rs6511720 functioned as an enhancer, whilst the rare alleles of both rs6511720 and rs57217136 increased *LDLR* promoter expression activity significantly by +29% and +24% (p = 0.026, and p = 0.002), respectively, when compared to the common alleles (Fig 3B).

### Identification of allele-specific transcription factor binding

MC-EMSAs were carried out to identify the specific TFs that bind to rs6511720 and rs57217136 variants. The results showed that the serum response element (SRE) is bound to a sequence around the protective allele (T) of rs6511720 (S2B Fig), while the retinoic acid receptor (RAR) and signal transducer and activator of transcription-1 (STAT1) are bound to a sequence around the C allele of rs57217136 (S2D Fig).

## Discussion

Several GWAS have reported that the *LDLR* SNP rs6511720 (G>T) is associated with lower plasma levels of LDL-C and a lower risk of CHD [19–21]. Data from the GLGC consortium showed that The *LDLR* rs6511720 minor (T, Forward strand) allele is carried by approximately 10% of the population and have confirmed the allele is protective, being associated with lower levels of LDL-C. This association is consistent and of sufficient strength to suggest that common variation in *LDLR* has implications for health, and that determining the precise molecular mechanism of the effect is relevant. The lead hit SNP has strong LD with 48 SNPs, thus to prioritize candidate variants, genome-wide maps of regulatory elements were used, which are useful resources to identify variants differentially affecting transcriptional activity. We found four promoter-proximal intron-1 variants (rs6511720, rs57217136, rs141787760 and rs60173709), which had strong chromatin signals in liver cells: these SNPs are in complete LD (r^2^ = 1) with the GWAS hit SNP. *In-silico* tools, MatInspector and HaploReg, showed different DNA-protein binding profiles of these SNPs, The *LDLR* SNP rs6511720 was predicted to be a strong enhancer, and the other three SNPs were predicted to be promoter-activators [48]. Although, the inconsistent prediction between *in-silico* tools prevented finding an interesting TF to test, the data shed light on candidate SNPs that have a strong regulatory profile, and which were worthy of further investigation using functional assays.

The *in-silico* findings were confirmed by EMSAs, where the four SNPs all showed allele-specific protein binding to the rare alleles. This finding suggested that these proteins may up-regulating *LDLR* expression. To determine whether the different genotypes have an influence on *LDLR* expression, gene reporter assays were used. Data from the luciferase reporter assays showed that *LDLR* intron-1 SNPs are indeed affecting *LDLR* gene expression, as the minor alleles of rs6511720 and rs57217136 showed significant higher expression, while rs141787760 and rs60173709 showed significant lower expression but no difference between the alleles. It is important to consider the combined effect on expression of these four SNPs, given that the rare alleles of all four are always present together. It is likely that the lowering effect of the minor alleles of rs141787760 and rs60173709 partially repress the raising effect of the rare alleles of rs6511720 and rs57217136 SNPs. If the combined effect of the minor alleles of four *LDLR* intron-1 SNPs was estimated by summation, we would predict that the haplotype will show ~ 29% higher expression. This would lead to up-regulation of LDL-R numbers on the surface of hepatocytes and this in turn would explain the lower levels of LDL-C in individuals carrying the rs6511720 minor allele haplotype.

The actual mechanism of this effect is only partially unraveled by our work. Both minor alleles of SNPs rs6511720 and rs57217136 are predicted to create enhancer-binding protein sites for TFs that would contribute to increased *LDLR* expression. MC-EMSAs showed rs6511720 minor allele (T) was bound to the serum response element (SRE) transcription factor. The SRE contains a binding site for serum response factors (SRF), which have a role in *LDLR* gene expression stimulation [49]. It also showed that rs57217136 was bound to RAR and STAT1. The Retinoic Acid receptor is a member of a family of nuclear receptor proteins actively involved in retinoic acid mediated transcriptional regulation of genes that controls lipid metabolism, through dimerization with other proteins to initiate transcription [50]. STAT1 is involved in lipid metabolism via JAK/STATs through several pathways. A phosphorylated STAT1 is inter-nuclear and binds to *sis*-inducible element (SIE) sequence in the promoter region and regulates gene expression [51, 52]^.^

### Limitations

Our results suggest that a cis-regulatory element near rs6511720 and rs57217136 SNPs acts in the liver cell line. However, in our study we did not examine other LD distal SNPs that may have a role in transcription regulation and therefore further studies are needed in this direction. In this study, we used luciferase reporter assays and EMSAs, which are techniques used to measure the difference of allelic expression and to determine DNA-protein interaction *in vitro*. An *in vitro* study may not fully represent what is occurring *in vivo*, where open chromatin structure and epigenetics have a potential role in gene regulation, and where transfection of a small fragment of DNA into a cell line cannot accurately reflect the natural situation. Finally, since we studied the effect of these SNPs only in liver cells we cannot determine whether or not they also influence LDLR gene expression in other tissues. However since the major site of expression of the LDLR is the liver, where clearance of LDL-C from the plasma occurs, this is not a major limitation.

In conclusion, integration of bioinformatics with GWAS disease-associated variants helps to elucidate gene-regulatory variants underlying association signals. Both rs6511720 and rs57217136 were identified as part of a cis-regulatory complex in a liver cell line that altered transcriptional activity through binding SRE, RAR, and STAT1. However, more studies are needed to define the spatial organization of the gene, which has a fundamental role in controlling gene expression [53–56], by the chromatin looping that brings enhancers and promoters into close spatial proximity to interact and initiate transcription. To identify the interaction between the functional SNPs and the *LDLR* promoter, chromosome conformation capture (3C) would be a useful technique [57, 58] or chromosome conformation capture carbon copy (5C) [59].

## Supporting Information

S1 Fig *LDLR* rs6511720 LD plot. A LD plot was generated using Locuszoom (http://csg.sph.umich.edu/locuszoom/). SNPs are plotted with the meta-analysis p value of LDL-C association (as–log_10_ values) as a function of genomic position. The lead SNP (rs6511720) is represented by a diamond, while LD SNPs are represented by circles. The LD SNPs are color coded to represent the r-squared between SNP and the putative associated variant, where red indicates a strong LD r^2^≥0.8 and dark blue indicates a weak LD r^2^≤0.2. A blue line indicates estimated recombination rates and dark blue arrows indicate gene annotations. LD and recombination rates are based on HapMap Phase II (CEU, YRI and JPT+CHB) or 1000 Genomes (CEU) and gene information from the UCSC browser.
(TIF)

S2 Fig DNA binding and expression of the transcription factors of the *LDLR* SNPs: rs6511720 and rs57217136. MC-EMSA analysis. Nuclear proteins from the Huh7 cell line were incubated with 7 cocktails of unlabelled DNA competitors (70 well-characterized DNA-binding proteins) for 15 minutes, then a 5’ end-biotinylated allele-specific probe was added. The multiplex competitors compete out any specific interactions with a labeled probe, eliminating or reducing any positive shift result. A) *LDLR* rs6511720 MC-EMSA for both alleles of the SNP, T allele (rare) specific bands were eliminated by cocktail 4. B) The single competitors from cocktail 4 (a) were run individually in a further EMSA, showing SRE resulted in competition. C) *LDLR* rs57217136 MC-EMSA for C allele, the C allele (rare) specific bands were eliminated by cocktail 4. D) The single competitors of the cocktail 4 (c) were run individually in a further EMSA, showing competitors RAR and STAT1 resulted in competition.
(TIF)

S1 Table Association of rs6511720 genotype and CHD risk in CARDIoGRAM and C4D. (PDF)

S2 Table Association of rs6511620 genotype and lipid treats from data in Global lipids genetics consortium (GLGC). (PDF)

S3 Table Predicted regulatory element and protein binding of LDLR selected SNPs. * minor allele.
(PDF)

## Abbreviations

CHD: coronary heart disease
EMSAs: electrophoretic mobility shift essays
FH: familial hypercholesterolemia
GWAS: genome-wide association study
LD: linkage disequilibrium
*LDLR*: Low-density lipoprotein receptor
LDL-C: Low-density lipoprotein cholesterol
MAF: minor allele frequency
MC-EMSA: multiplex competitor-EMSAs
RAR: retinoic acid receptor
SNP: single nucleotide polymorphism
SRE: serum response element
STAT1: signal transducer and activator of transcription 1
