# Supplementary figures and images for: Identification of the Functional Variant(s) that Explain the Low-Density Lipoprotein Receptor (LDLR) GWAS SNP rs6511720 Association with Lower LDL-C and Risk of CHD
Source: PLoS One. 2016 Dec 14;11(12):e0167676. doi: 10.1371/journal.pone.0167676 (PMC5156384; doi:10.1371/journal.pone.0167676)

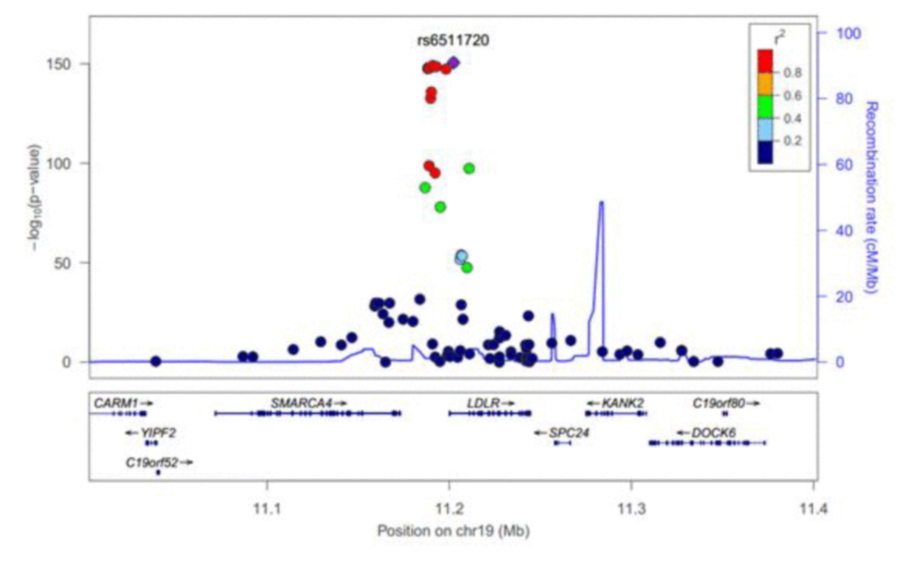

Supplement: S1 Fig — A LD plot was generated using Locuszoom (http://csg.sph.umich.edu/locuszoom/). SNPs are plotted with the meta-analysis p value of LDL-C association (as–log10 values) as a function of genomic position. The lead SNP (rs6511720) is represented by a diamond, while LD SNPs are represented by circles. The LD SNPs are color coded to represent the r-squared between SNP and the putative associated variant, where red indicates a strong LD r2≥0.8 and dark blue indicates a weak LD r2≤0.2. A blue line indicates estimated recombination rates and dark blue arrows indicate gene annotations. LD and recombination rates are based on HapMap Phase II (CEU, YRI and JPT+CHB) or 1000 Genomes (CEU) and gene information from the UCSC browser. (TIF) [file pone.0167676.s001.tif]

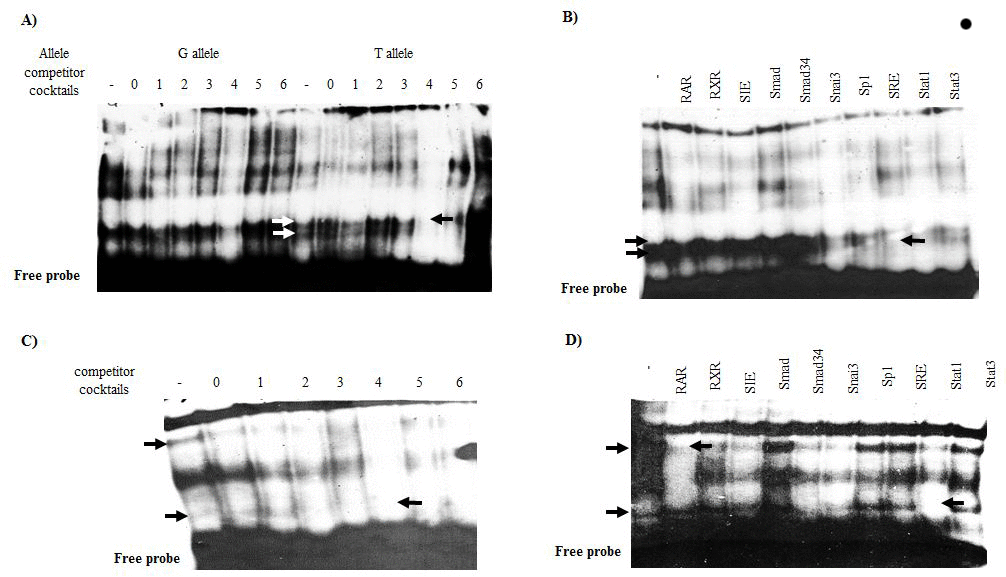

Supplement: S2 Fig — MC-EMSA analysis. Nuclear proteins from the Huh7 cell line were incubated with 7 cocktails of unlabelled DNA competitors (70 well-characterized DNA-binding proteins) for 15 minutes, then a 5’ end-biotinylated allele-specific probe was added. The multiplex competitors compete out any specific interactions with a labeled probe, eliminating or reducing any positive shift result. A) LDLR rs6511720 MC-EMSA for both alleles of the SNP, T allele (rare) specific bands were eliminated by cocktail 4. B) The single competitors from cocktail 4 (a) were run individually in a further EMSA, showing SRE resulted in competition. C) LDLR rs57217136 MC-EMSA for C allele, the C allele (rare) specific bands were eliminated by cocktail 4. D) The single competitors of the cocktail 4 (c) were run individually in a further EMSA, showing competitors RAR and STAT1 resulted in competition. (TIF) [file pone.0167676.s002.tif]
