# Supplementary material for: Identification of the Functional Variant(s) that Explain the Low-Density Lipoprotein Receptor (LDLR) GWAS SNP rs6511720 Association with Lower LDL-C and Risk of CHD
Source: PLoS One. 2016 Dec 14;11(12):e0167676. doi: 10.1371/journal.pone.0167676 (PMC5156384; doi:10.1371/journal.pone.0167676)
Supplement: S1 Table — (PDF) [file pone.0167676.s003.pdf]

S1 Table. Association of rs6511720 genotype and CHD risk in CARDIoGRAM and C4D

SNP(allele)	log(OR) with CHD per-allele	SE (log(OR)	Sample numbers
**rs6511720 (T~minor allele) CARDIoGRAM**	- 0.1253	0.03321	8948cases 4747 controls
**rs6511720 (T~minor allele) C4D**	- 0.1082	0.0287	15393 cases 15036 controls
**fixed effects meta-analysis**	- 0.1155	0.0217	24341 cases 19783 controls
