# Supplementary material for: Identification of the Functional Variant(s) that Explain the Low-Density Lipoprotein Receptor (LDLR) GWAS SNP rs6511720 Association with Lower LDL-C and Risk of CHD
Source: PLoS One. 2016 Dec 14;11(12):e0167676. doi: 10.1371/journal.pone.0167676 (PMC5156384; doi:10.1371/journal.pone.0167676)
Supplement: S2 Table — (PDF) [file pone.0167676.s004.pdf]

**S2 Table: Association of rs6511620 genotype and lipid treats from data in Global lipids genetics consortium (GLGC)**

| | Reference allele | Beta | Se | p-value | Sample size |
|---|---|---|---|---|---|
| **TC** | T | -0.1851 | 0.0059 | $5.43X10^{-202}$ | 184764 |
| **LDL-C** | T | -0.2209 | 0.0061 | $3.85\ X10^{-262}$ | 170608 |
| **HDL-C** | T | 0.0249 | 0.0057 | $6.32\ X10^{-005}$ | 184617 |
| **TG** | T | -0.0084 | 0.0056 | 0.1043 | 175280 |
