# Supplementary material for: Identification of the Functional Variant(s) that Explain the Low-Density Lipoprotein Receptor (LDLR) GWAS SNP rs6511720 Association with Lower LDL-C and Risk of CHD
Source: PLoS One. 2016 Dec 14;11(12):e0167676. doi: 10.1371/journal.pone.0167676 (PMC5156384; doi:10.1371/journal.pone.0167676)
Supplement: S3 Table — * minor allele. (PDF) [file pone.0167676.s005.pdf]

S3 Table. Predicted regulatory element and protein binding of *LDLR* selected SNPs

SNP IDs	Position (hg19)	Regulatory element [30]	Allele	Predicted protein binding (Haploreg)	Predicted protein binding (MatInspector)
**rs6511720**	11202306	Strong Enhancer	G	-	BLMP
			T*	-	GATA1, SNAP/PSE
**rs141787760**	11202194	Active Promoter	C	CDP	-
			-*	CHD2, CTCF, ELF1, Egr-1, SP1, Zic, and p300	-
**rs60173709**	11201988	Active Promoter	T	EBF, Myf, Pax-5	-
			-*	GR and MAZ	-
**rs57217136**	11201124	Active Promoter	T	FOXA1, FOXA2, and SREBP1	-
			C*	SP1	OAZF/ROAZ

* Minor allele
